# The disulfidptosis-related signature predicts prognosis and immune features in glioma patients

**DOI:** 10.1038/s41598-023-45295-w

**Published:** 2023-10-20

**Authors:** Xiong Wang, Jijun Yang, Fengjie Yang, Ketao Mu

**Affiliations:** 1 Department of Laboratory Medicine, Tongji Hospital, Tongji Medical College, Huazhong University of Science and Technology, Wuhan, China; 2 Department of Dermatology, Tongji Hospital, Tongji Medical College, Huazhong University of Science and Technology, Wuhan, China; 3 Department of Pediatrics, Tongji Hospital, Tongji Medical College, Huazhong University of Science and Technology, Wuhan, China; 4 Department of Radiology, Tongji Hospital, Tongji Medical College, Huazhong University of Science and Technology, Wuhan, China

**Keywords:** Prognostic markers, Cancer genomics

## Abstract

Glioma is the most common primary malignant tumor in the central nervous system. Disulfidptosis is a recently identified programmed cell death in tumor cells overexpressing SLC7A11 under glucose starvation. Clinical prognostic significance of disulfidptosis has been reported in several tumors, and in this study, we explored the correlation of disulfidptosis with clinical prognosis, immune cell infiltration, and immunotherapy response in glioma. A total of 1592 glioma patients were included in this study, including 691 glioma patients from The Cancer Genomic Atlas (TCGA), 300 patients with from the Chinese Glioma Genomic Atlas (CGGA) array, 325 patients from CGGA sequencing, and 276 patients from Gene Expression Omnibus (GEO) GSE16011. R software (V4.2.2) and several R packages were applied to develop the risk score model and correlation calculation and visualization. Three disulfidptosis-related genes, LRPPRC, RPN1, and GYS1, were screened out and applied to establish the risk score model. Low-risk patients exhibit favorable prognosis, and the disulfidptosis-related signature significantly correlated with clinicopathological properties, molecular subtypes, and immunosuppressive microenvironment of glioma patients. We developed a disulfidptosis-related risk model to predict the prognosis and immune features in glioma patients, and this risk model may be applied as an independent prognostic factor for glioma.

## Introduction

Glioma is the most common primary malignant tumor in the central nervous system (CNS), accounting for 47.7% of all malignancies in the CNS^1^. Glioma is divided into lower‐grade gliomas (LGG, WHO grade II and III) and glioblastoma (GBM, WHO grade IV). GBM patients exhibit more unsatisfactory prognosis than LGG patients. The general median survival time of GBM patients was 12 months after surgery and radiotherapy, while the survival time of LGG patients varied from 1 to 15 years^2^. The current therapeutic interventions including surgery, chemo‐ and radio‐ therapy, fails to improve the prognosis of glioma patients especially for the GBM patients, indicating the need for early detection and novel intervention for glioma patients^3^. Previous cancer genetics have revealed several molecular markers in glioma, such as isocitrate dehydrogenase (IDH) mutations, O-6-methylguanine-DNA methyltransferase (MGMT) promoter methylation, and 1p19q codeletion, and these molecular markers were correlated with favorable prognosis of glioma patients^4^. However, many glioma patients still have minimal responses to these molecular markers targeted therapies, suggesting the need to investigate novel biomarkers for glioma prognosis and treatment prediction.

Programmed cell death (PCD) is induced intentionally accompanied by numerous controlled steps resulting in well-programmed self-destruction during development, including apoptosis, cuproptosis, ferroptosis, pyroptosis, and PANoptosis^5^. Dysregulation of PCD correlates with the development, metastasis, mortality, and recurrence of tumors^6^. Recently, Liu et al. reported a novel metabolic-related PCD, disulfidptosis, which is induced by excessive accumulation of disulfide in glucose-starved tumor cells overexpressing Solute Carrier Family 7 Member 11 (SLC7A11)^7^. SLC7A11 (also known as xCT) is upregulated in multiple cancers, which imports cystine for glutathione generation and antioxidant defense to block ferroptosis and necroptosis^8^. Under glucose starvation, SLC7A11 overexpression mediated cystine uptake induces nicotinamide adenine dinucleotide phosphate hydrogen (NADPH) depletion, intracellular disulfide accumulation, and ultimate disulfidptosis^7^. Disulfidptosis is correlated with prognosis, the tumor microenvironment (TME) and anti-tumor immunity in several tumors, including thyroid carcinoma, bladder cancer, and lung adenocarcinoma^9–11^, however, the association between disulfidptosis and prognosis, TME, and immune therapy response in glioma is unclear.

In the study, we comprehensively explore the role of disulfidptosis-related genes in the prognosis, TME landscapes, and immune therapy in glioma. We developed and validated a disulfidptosis-related prognostic model with good performance in predicting prognosis and response to immunotherapy across four independent cohorts.

## Materials and methods

### Data collection

The Cancer Genome Atlas (TCGA) LGG and GBM datasets were downloaded using the TCGAbiolinks (v2.26.0)^12^. The Chinese Glioma Genome Atlas (CGGA) (mRNAseq_325) and CGGA_array datasets (mRNA-array_301) were downloaded from CGGA website (http://www.cgga.org.cn)^13–17^. The expression data of GSE16011 was downloaded from the Gene Expression Omnibus (GEO) database (https://www.ncbi.nlm.nih.gov/geo/query/acc.cgi?acc=GSE16011), and the clinical data was downloaded from the supplementary data of their published work^18^. These data were cleaned and combined using the tinyarray (v2.2.9) R package.

### Immunofluorescence staining

The subcellular distribution of these three genes in glioma cell line U-251MG were analyzed using immunofluorescence staining data from HPA database (http://www.proteinatlas.org), and the results showed that LRPPRC, RPN1, and GYS1 were in mitochondria, cytosol, and microtubules, respectively.

### Disulfidptosis-related risk signature construction and validation

A total of ten disulfidptosis-related genes were selected from previous studies, including GYS1, OXSM, NDUFS1, LRPPRC, NDUFA11, NUBPL, NCKAP1, RPN1, SLC3A2 and SLC7A11^7^. Univariate Cox regression and Kaplan–Meier (KM) analyses were performed with survival (v3.5-3) R package to screen disulfidptosis-related genes significantly correlated with the overall survival (OS). Genes with p values < 0.05 in both analyses were selected for multivariate Cox regression analysis, and TCGA dataset was used as the training cohort. The results of multivariate Cox regression analysis and the KM plots were shown using the survminer (v0.4.9) R package.

### Time dependent receiver operating characteristic (ROC) analysis

Time dependent receiver operating characteristic (ROC) curves were calculated using the timeROC (v.0.4) R package to predict the 1-year, 3-year, and 5-year outcomes of glioma patients.

### Functional enrichment analysis

The significantly different Kyoto Encyclopedia of Genes and Genomes (KEGG) pathways and Gene Ontology (GO) biological processes between high-risk and low-risk glioma patients were analyzed using the GSVA (v1.46.0) R package^19^, and the heatmap was shown with pheatmap (v1.0.12) R package.

### Evaluation of immune cell fractions

Molecular marker genes of 28 types of immune cells were extracted from previous published work^20^. The immune cell composition was calculated using the ssgsea method of the GSVA (v1.46.0) R package.

### Immune subtype analysis

Tumor samples could be divided into six subtypes: C1 (Wound Healing), C2 (IFN‐γ Dominant), C3 (Inflammatory), C4 (Lymphocyte Depleted), C5 (Immunologically Quiet), and C6 (TGF‐β Dominant)^20^. The immune subtype analysis was performed using the ImmuneSubtypeClassifier (v0.1.0) R package.

### Figure and plot generation

The venn plot was drawn by tinyarray (v2.2.9) R package. The forest plot and survival KM plot were drawn by survminer (v0.4.9) R package. The timeROC curve was drawn by ggplot2 (v3.4.3) R package. The beeswarm plot was drawn by ggbeeswarm (v0.7.2) R package. The box plot and scatter plot were drawn by ggpubr (v0.6.0) R package.

## Results

### Construction of risk model using three disulfidptosis-related genes

**Figure 1:**
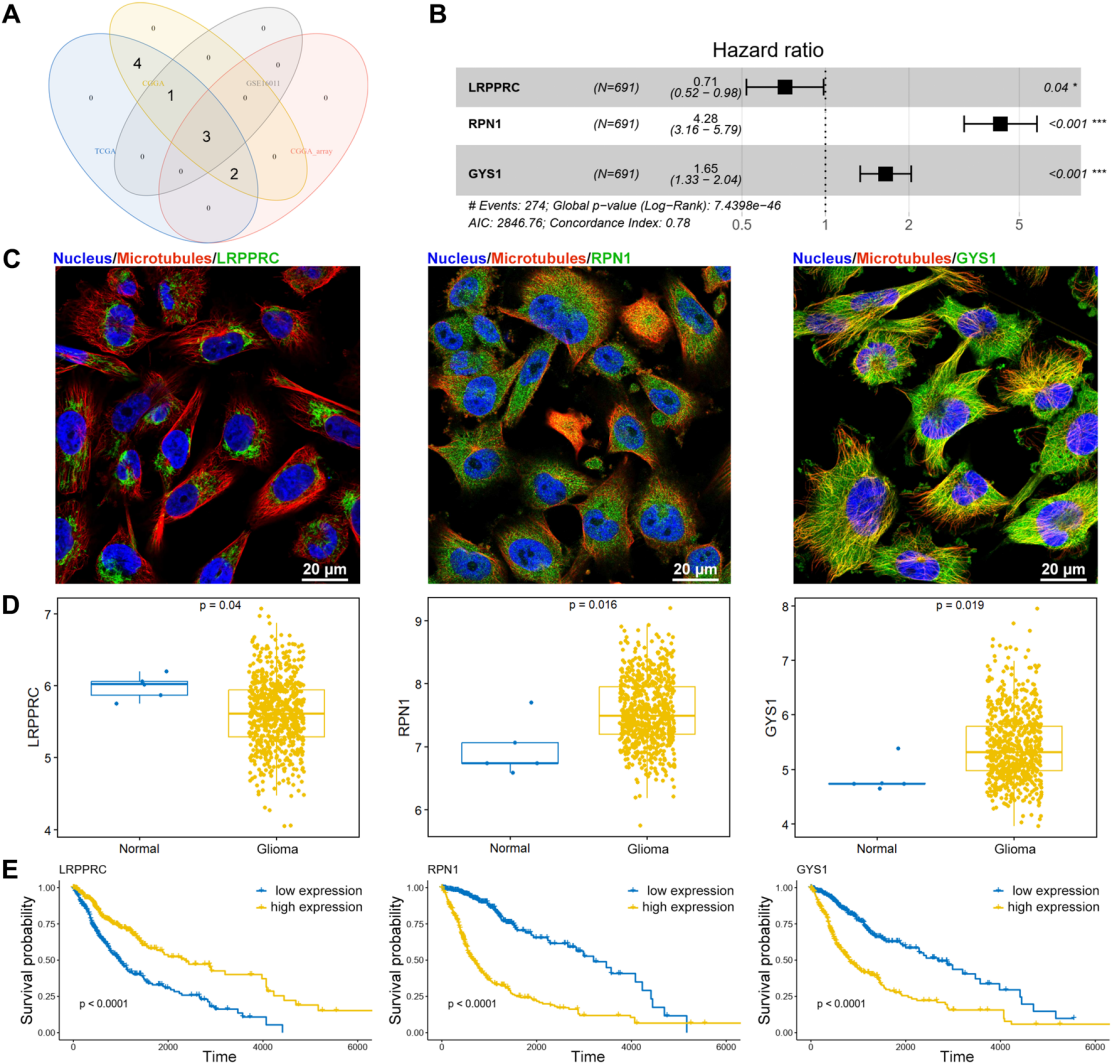
Construction of risk model using three disulfidptosis-related genes. (**A**) Intersected genes correlated with prognosis of glioma in both univariable Cox regression and KM survival analyses from the TCGA, CGGA, CGGA‐array, and GSE16011 datasets. (**B**) The Cox Proportional Hazards Model was used to construct the risk model using LRPPRC, RPN1, and GYS1 genes. (**C**) Immunofluorescence staining of LRPPRC (antibody: HPA036409), RPN1 (antibody: HPA051520), and GYS1 (antibody: HPA041598) in U-251MG glioma cells from HPA database. (**D**) The expression levels of LRPPRC, RPN1, and GYS1 between glioma (LGG + GBM) and normal control from TCGA were compared and displayed using boxplot. LRPPRC was decreased in glioma, while RPN1 and GYS1 were increased in glioma tissues. (**E**) K-M survival curves for LRPPRC, RPN1, and GYS1 genes showed that high expression of LRPPRC had better OS, while high expression of RPN1 and GYS1 had worse OS.

A total of ten disulfidptosis-related genes were selected from previous studies, including GYS1, OXSM, NDUFS1, LRPPRC, NDUFA11, NUBPL, NCKAP1, RPN1, SLC3A2 and SLC7A11^7^. Both univariable Cox regression and KM survival analyses were performed to screen disulfidptosis-related genes significantly correlated to the prognosis of glioma in all four datasets. A total of three significantly correlated disulfidptosis-related genes were identified, including LRPPRC, RPN1, and GYS1 (Fig. 1A, Tables S1, 2). The AIC method of Cox Proportional Hazards Model was used to construct the risk model (Fig. 1B, Table 1). The subcellular distribution of these three genes in glioma cell line U-251MG were analyzed using immunofluorescence staining data from HPA database (http://www.proteinatlas.org), and the results showed that LRPPRC, RPN1, and GYS1 were in mitochondria, cytosol, and microtubules, respectively (Fig. 1C). We explored their expression in dataset from TCGA and found that LRPPRC was downregulated, while RPN1 and GYS1 were upregulated in TCGA glioma dataset (Fig. 1D). Furthermore, the KM survival plots showed that high expression of LRPPRC was positively correlated with good prognosis, while high expression of RPN1 and GYS1 was positively correlated with poor prognosis (Fig. 1E). These data suggest that these three disulfidptosis-related genes were dysregulated in glioma and were associated with the prognosis of glioma.

**Table 1:** The Cox Proportional Hazards Model using three disulfidptosis-related genes.

Gene	coef	exp(coef)	se(coef)	z	p value
LRPPRC	− 0.3358	0.7148	0.1634	− 2.055	0.0398
RPN1	1.4536	4.2785	0.1543	9.422	< 2e−16
GYS1	0.5002	1.6491	0.1081	4.627	3.71e−06

### Evaluation of the disulfidptosis-related risk signature

**Figure 2:**
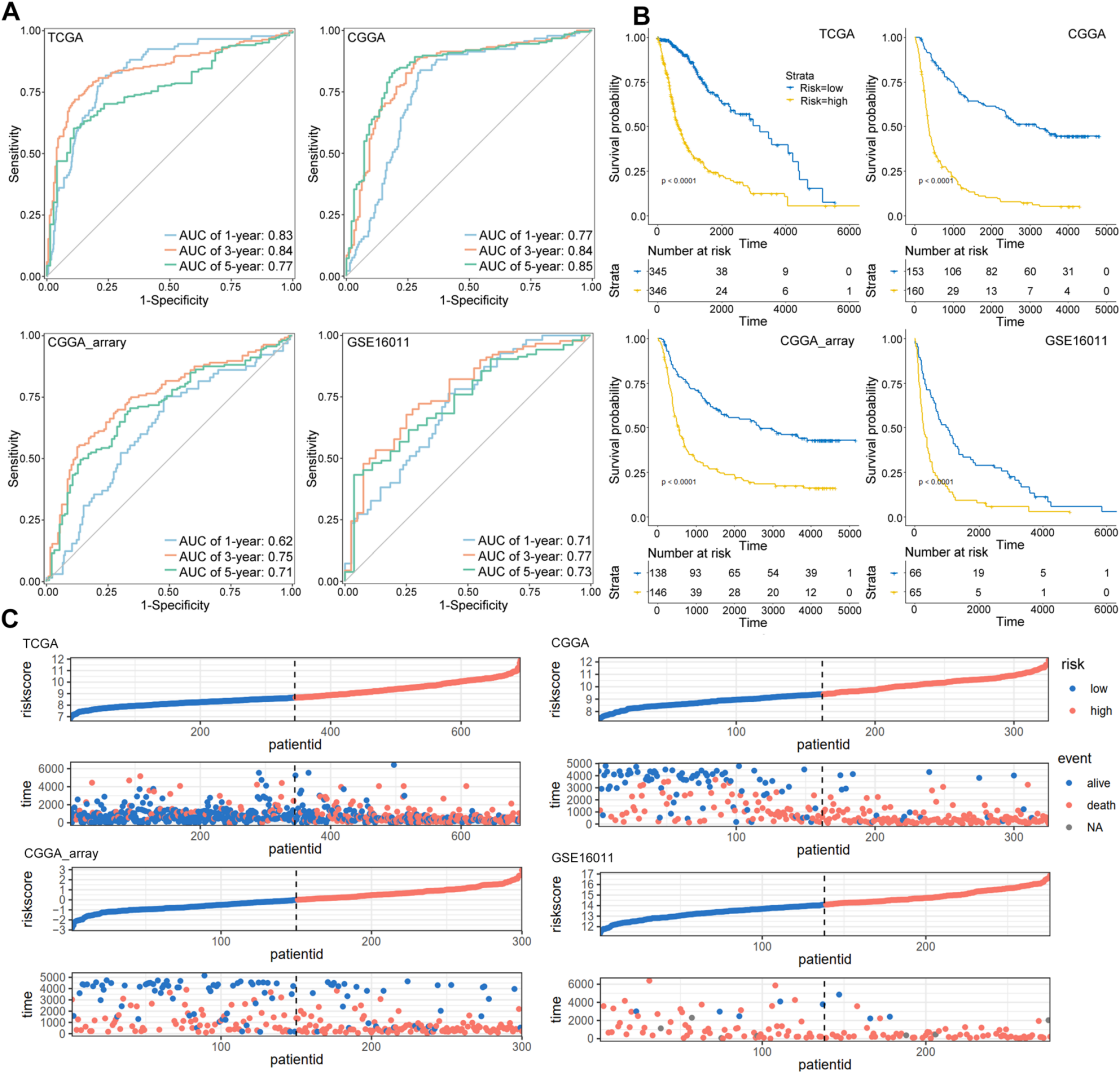
Evaluation of the disulfidptosis-related risk signature. (**A**) The 1-, 3-, and 5-year time-dependent OS ROC curves of the disulfidptosis-related risk signature in the four datasets were calculated using the timeROC R package. (**B**) The KM survival plots showed that patients with low-risk had better OS. (**C**) The risk score distribution and outcome of glioma patients in all four datasets.

The TCGA glioma dataset was used to construct the risk model, and the risk score was calculated using the following formula: risk score = (− 0.3358 × LRPPRC expression) + (1.4536 × RPN1 expression) + (0.5002 × GYS1 expression). The prognostic prediction potential of the disulfidptosis-related risk signature was examined with time-dependent ROC curves. The results showed that this risk signature had better AUC values in 3- and 5-year OS prediction and the CGGA dataset had the best prediction potential with an AUC value of 0.85 for 5-year OS (Fig. 2A). Patients were grouped into high and low-risk groups using the cutoff of median risk score value, and the KM survival plots showed that patients with low-risk had better OS in all four datasets (Fig. 2B). The risk score distribution and outcome of patients showed that most alive patients were in the low-risk group, while most dead patients had higher risk scores (Fig. 2C), suggesting the accurate prediction potential of the disulfidptosis-related risk signature for glioma patients.

### Correlation between the disulfidptosis-related signature and clinicopathological features

**Figure 3:**
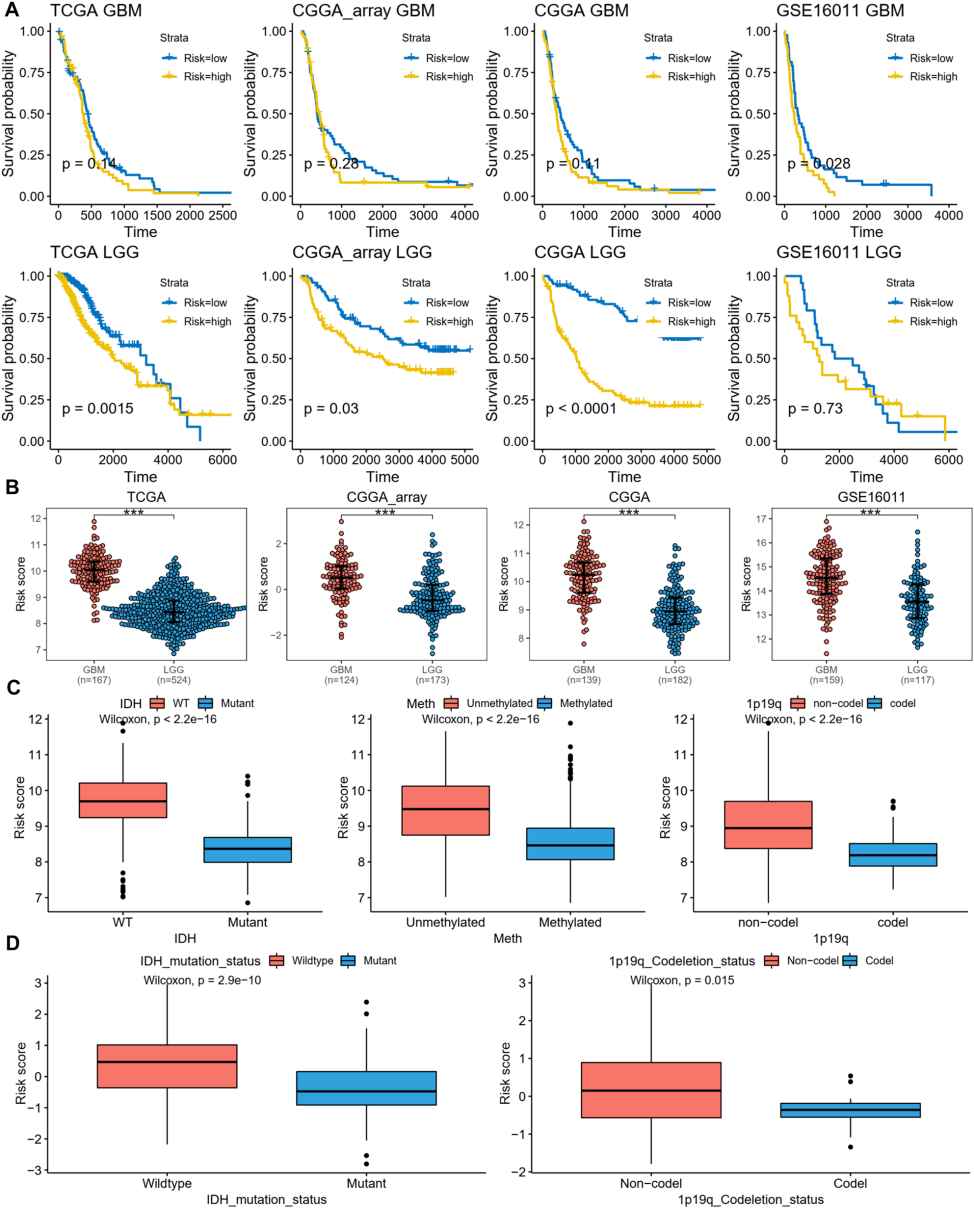
Correlation between the disulfidptosis-related signature and clinicopathological features. (**A**) The KM survival plots showed the survival probability of the disulfidptosis-related signature risk score and OS of GBM and LGG respectively. (**B**) The beeswarm plot showed the difference of risk scores between GBM and LGG patients in all four datasets. (**C**) The boxplot showed the difference of risk scores between IDH wildtype (WT) and mutant, MGMT promoter unmethylated and methylated, 1p19q non-codeletion and codeletion patients from TCGA dataset. (**D**) The boxplot showed the difference of risk scores between IDH wildtype and mutant, 1p19q non-codeletion and codeletion patients from CGGA dataset. ***p < 0.001.

Since the disulfidptosis-related signature showed remarkable correlation with OS of glioma patients and harbored accurate prediction potential, we further explored the correlation between the disulfidptosis-related signature and clinicopathological features considering malignancy grade, IDH mutation, MGMT promoter methylation, and 1p19q codeletion status. The KM survival plots showed that patients with low-risk had better OS of LGG patients in TCGA, CGGA_array, CGGA datasets, while had better OS of GBM patients in GSE16011 dataset (Fig. 3A). In all four datasets, we found that GBM patients, representing higher grade, had higher risk scores than LGG patients (Fig. 3B). In TCGA dataset, we observed that IDH wildtype, MGMT promoter unmethylated, and 1p19q non-codeletion patients had higher risk scores (Fig. 3C), and similar results were found in CGGA dataset (Fig. 3D). These results indicate that the disulfidptosis-related signature is significantly correlated with clinicopathological features of glioma patients.

### Functional annotation of the disulfidptosis-related signature

**Figure 4:**
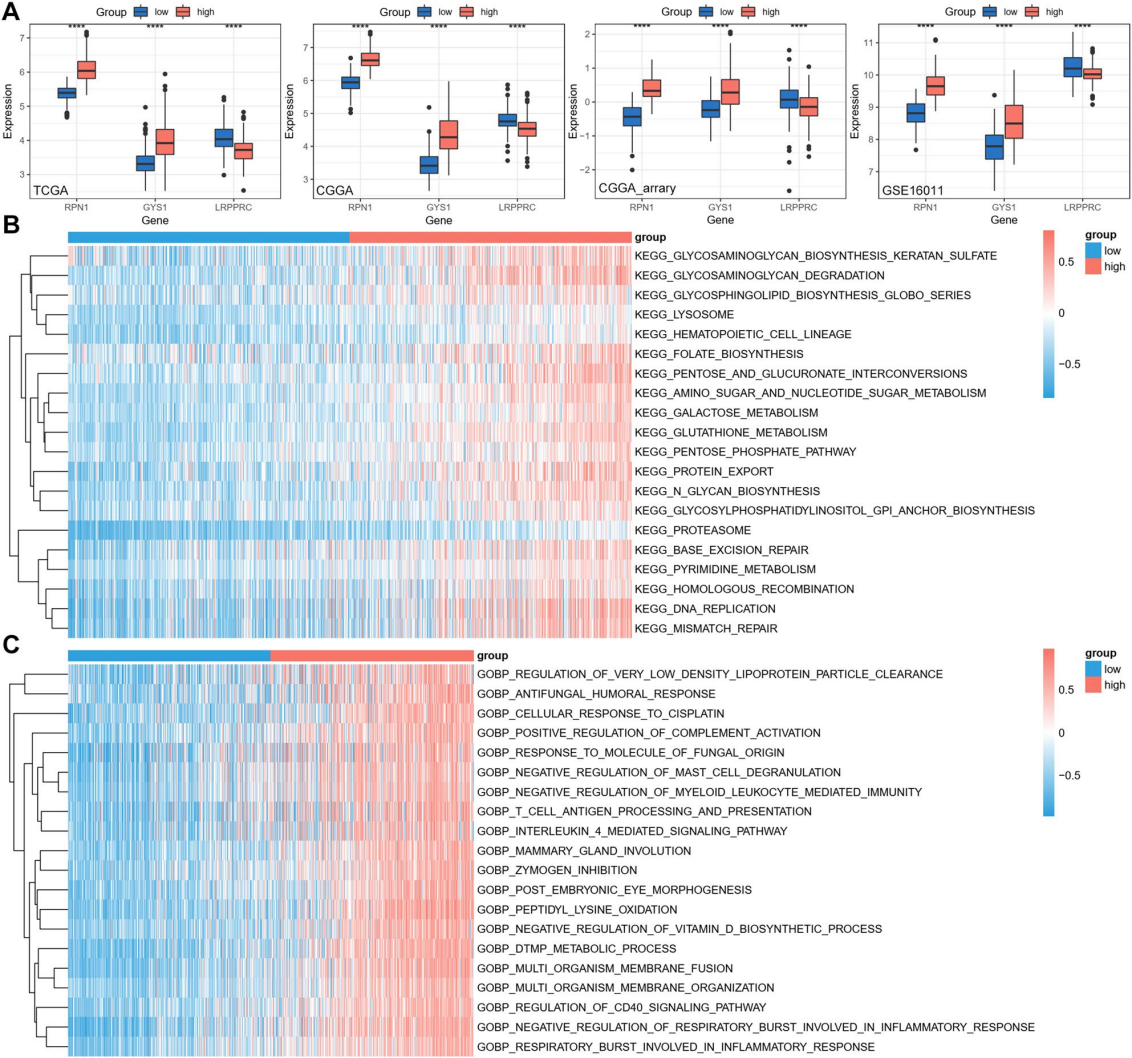
Functional annotation of the disulfidptosis-related signature. (**A**) The expression of LRPPRC, RPN1, and GYS1 between low and high-risk glioma patients from all four datasets. (**B**) The top 20 KEGG pathways enriched in the high‐risk glioma patients from TCGA dataset. (**C**) The top 20 biological processes enriched in the high‐risk glioma patients from TCGA dataset. ****p < 0.0001.

We further investigated the functional annotation of the disulfidptosis-related signature concerning the KEGG pathways and biological processes. The expression of the three disulfidptosis-related genes, LRPPRC, RPN1, and GYS1, was examined, and the results showed that RPN1 and GYS1 were increased in high-risk group while LRPPRC was decreased in high-risk group in all four datasets (Fig. 4A). The differences of KEGG pathways and biological processes between low and high-risk groups were compared using the GSVA R package. The significantly different KEGG pathways enriched in high-risk glioma patients involved sugar metabolisms, including glycosaminoglycan, glycosphingolipid, amino and nucleotide sugar (Fig. 4B). The biological processes enriched in high-risk glioma patients involved immune responses, including complement activation, mast cell degranulation, and antigen processing and presentation (Fig. 4C). Disulfidptosis occurred under glucose starvation, and these results suggest that the three disulfidptosis-related gene may regulate disulfidptosis through sugar metabolisms.

### Correlation between the disulfidptosis-related signature and immune features

**Figure 5:**
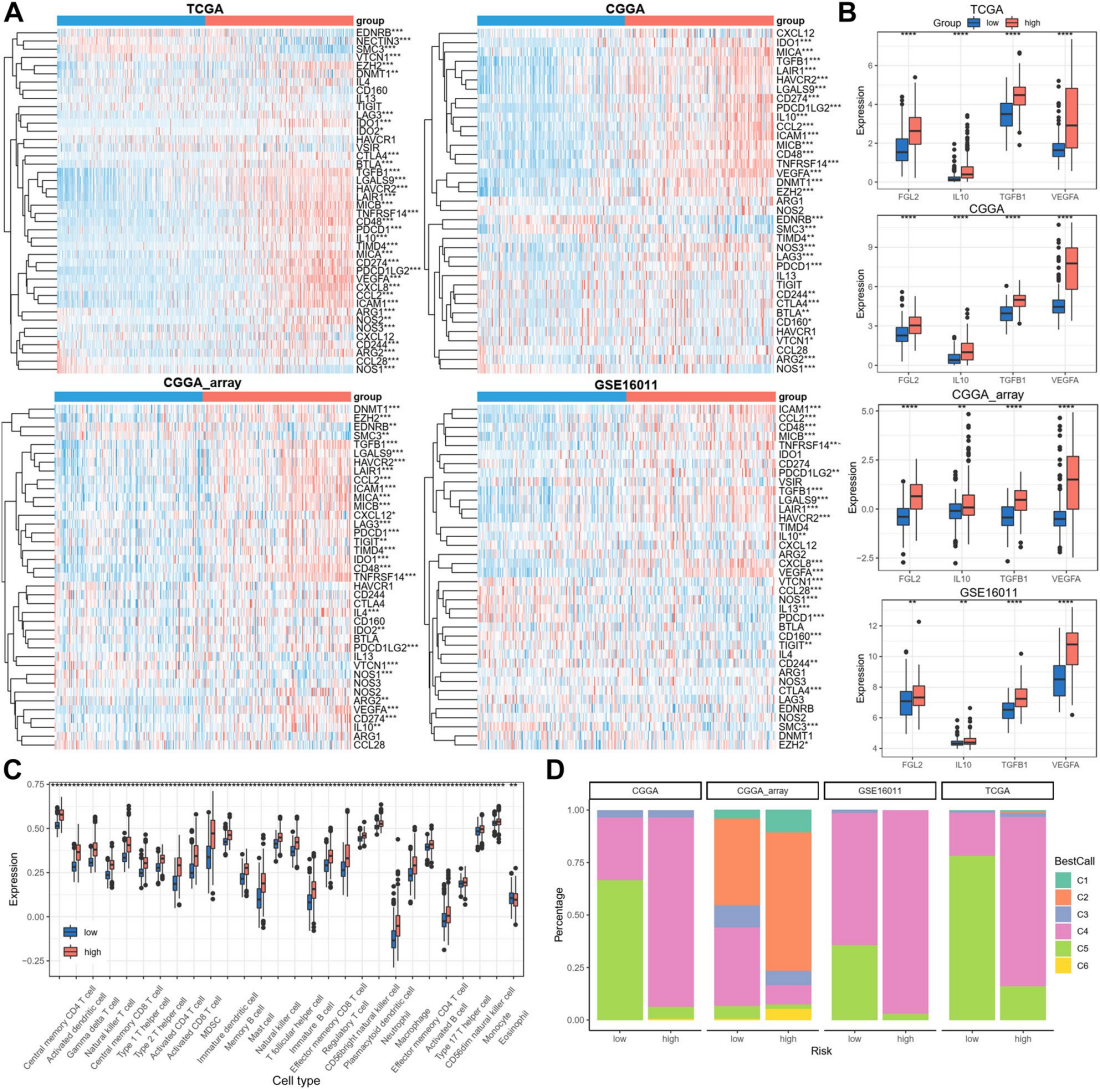
Correlation between the disulfidptosis-related signature and immune features. (**A**) Expression of the cancer‐immunity cycle inhibitory genes between low‐ and high‐risk groups. (**B**) Expression of FGL2, IL10, TGFB1, and VEGFA between low‐ and high‐risk groups. (**C**) Immune cell ratio between low‐ and high‐risk groups from TCGA dataset. (**D**) Distribution of immune subtypes. **p < 0.01, ****p < 0.0001.

As immune response biological processes were differentially enriched in low and high-risk glioma patients revealed above, we studied the correlation between the disulfidptosis-related signature and immune features. Anti-cancer immune response consists of several stepwise events named the cancer-immunity cycle, involving several immune cells and molecules. An inhibitory gene list of the cancer-immunity cycle was downloaded TIP—Tracking Tumor Immunophenotype database^21^, and most of these inhibitory genes were upregulated in high-risk group from all four datasets (Fig. 5A). FGL2, IL10, TGFB1, and VEGFA were secreted immunosuppressive molecules in glioma and were consistently upregulated in high-risk group from all four datasets (Fig. 5B). The immune cell infiltration was analyzed between low and high-risk groups, most immune cells were enriched in high-risk groups including both oncogenic and immunosuppressive cells (Fig. 5C). The glioma patients were divided into six immune subtypes. C4 (Lymphocyte Depleted) and C5 (Immunologically Quiet) accounted for the majority, and C4 subtype representing a high M2 response showed higher ratio in high-risk groups (Fig. 5D). These data indicate that high disulfidptosis-related risk score represents immunosuppressive status in glioma.

**Figure 6:**
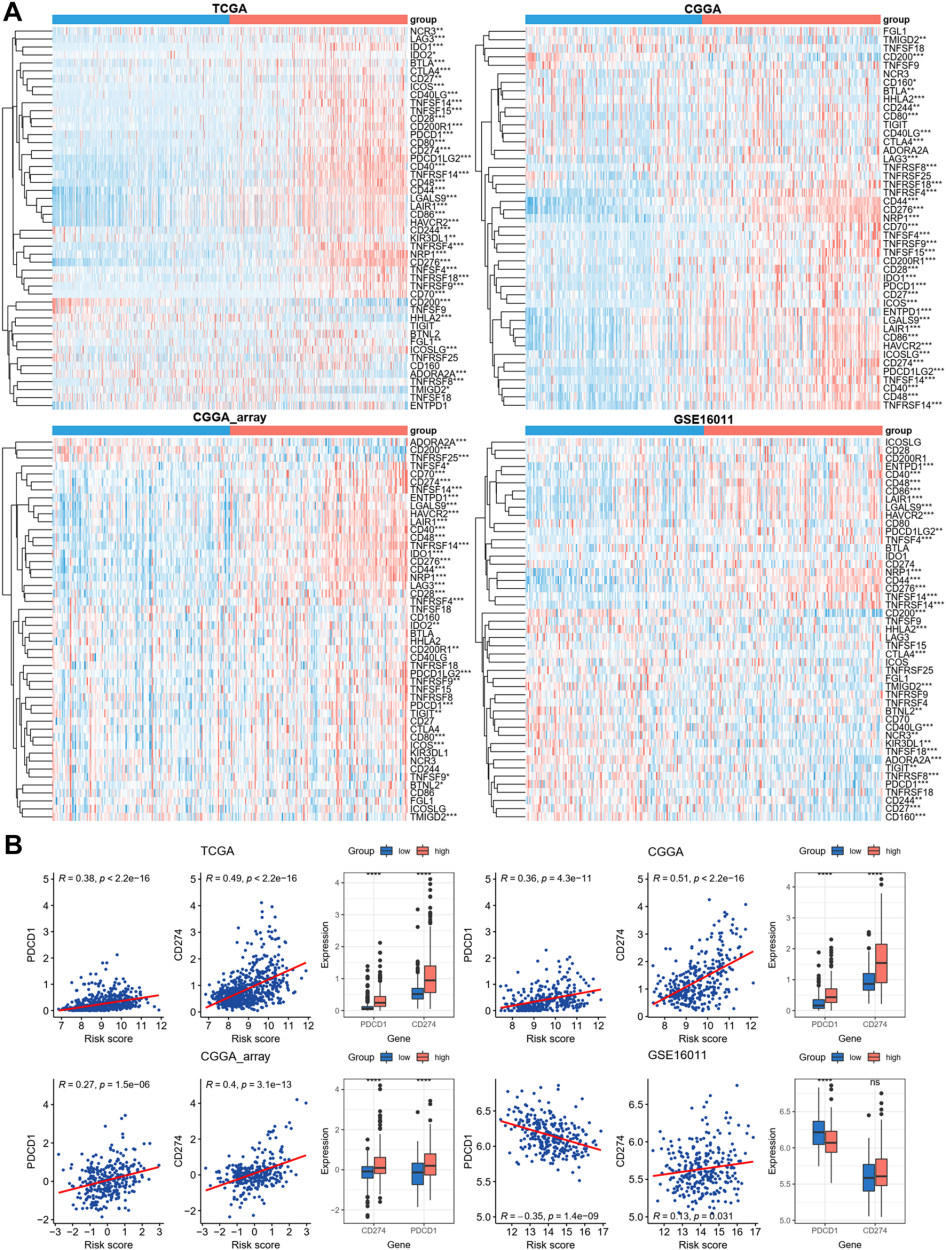
Correlation between the disulfidptosis-related signature and immune checkpoint genes. (**A**) Expression of immune checkpoint genes between low‐ and high‐risk groups. (**B**) Expression of PD-1 (PDCD1) and PD-L1 (CD274) between low‐ and high‐risk groups. Correlation between disulfidptosis-related signature risk score and the expression of PD-1 and PD-L1. ****p < 0.0001.

Moreover, the expression of immune checkpoints indicates the response of immunotherapy, and we also found that most immune checkpoint genes were upregulated in high-risk groups (Fig. 6A). PD-1 (PDCD1) and PD-L1 (CD274) have been widely used in immune checkpoint inhibitor immunotherapy, and the results showed that both PD-1 and PD-L1 were increased in high-risk groups, furthermore, disulfidptosis-related risk score was positively correlated with the expression of PD-1 and PD-L1 (Fig. 6B). These results suggest that disulfidptosis‐related risk signature can predict the immune features of glioma.

## Discussion

Due to the metabolic imbalance and rapid proliferation, buffering reactive oxygen species (ROS), acidic extracellular pH, hypoxia, and nutrient deprivation to thrive in the tumor microenvironment are the main challenges for cancers. Metabolic reprogramming is an essential hallmark of cancer cells, which results in a high dependence of specific nutrients or metabolites and promotes the survival of cancer cells^22^. For instance, to mitigating the damaging effects of ROS, cancer cells upregulate the expression of SLC7A11, a cystine-glutamate antiporter, increase the import of cystine to generate cysteine and glutathione. However, under the glucose starvation situation, the cystine accumulates in cancer cells due to the disrupted conversion into cysteine caused by the insufficient supply of NADPH produced from glucose. Eventually, the accumulation of cystine causes aberrant disulfide bonding to the actin cytoskeleton, leading to disulfidptosis^7^. Liu et al. identified several genes remarkably associated with disulfidptosis via whole-genome CRISPR-Cas9 screen, including SLC7A11 and its chaperone SLC3A2, and various components of mitochondrial oxidative phosphorylation system (such as NDUFA11, NDUFS1, LRPPRC, and NUBPL)^7^. In this study we comprehensively studied the diagnostic and immunologic potential of these disulfidptosis-related genes using a risk score model. We established a 3-gene risk model which exhibited good performance in predicting both prognosis and immunotherapeutic response.

LRPPRC, RPN1, and GYS1 were identified as survival related disulfidptosis genes via univariable Cox regression and KM survival analyses among all the four datasets, and a risk score model was constructed using these three disulfidptosis related genes. LRPPRC is involved in mitochondrial oxidative phosphorylation, RPN1 locates in the endoplasmic reticulum, and GYS1 is a glycogen synthase. Immunofluorescence staining revealed that LRPPRC, RPN1, and GYS1 locate in mitochondria, cytosol, and microtubules, respectively. RPN1 and GYS1 were upregulated in glioma and were negatively correlated with favorable outcome, while LRPPRC1 was downregulated in glioma and positively correlated with favorable outcome. RPN1 encodes a type I integral membrane protein located in endoplasmic reticulum and is involved in the regulation of dolichyl-diphosphooligosaccharide-protein glycotransferase activity. Higher RPN1 somatic mutation was found in germline ALK variant glioma patients compared with germline ALK wildtype patients IDH wildtype glioma^23^. GYS1 is one of the main regulators of glycogen synthesis, and GYS1 inhibition causes glycogen accumulation in glioblastoma cells, leading to proliferation and migration suppression and formation of ROS, indicating GYS1 inhibition may be a promising therapeutic target for glioma^24,25^. These studies suggest that the three survival related disulfidptosis genes may also contribute to the progression of glioma and become the potential therapeutic targets.

Risk score is a convenient and widely used method for the construction of meaningful signatures. Our model was built with disulfidptosis‐related risk scores which showed both accurate prediction of the prognosis of glioma patients and excellent distinction for several glioma molecular subtypes including grade, IDH mutation, MGMT promoter methylation, and 1p19q codeletion status. The smallest AUC value of 3-year survival was 0.75 found in CGGA_array dataset, indicating good performance for short-term prediction. The AUC values of long‐term (5‐year) survival prediction were larger than 0.7 with the largest AUC of 0.85 in CGGA dataset. Patients were further divided into high-risk and low-risk subgroups using the median risk score as cutoff value, and low-risk patients showed better prognosis in all four datasets. Moreover, patient distribution also revealed that the alive patients were mainly found in low-risk subgroup, while dead patients were mainly distributed in high-risk subgroup. LGG is low grade glioma (WHO grade II and III), which may progress to the high grade (IV) GBM glioma. Consistently, LGG patients had lower risk scores than GBM patients.

IDH play essential roles in Krebs cycle and cellular homoeostasis via catalyzing the oxidative decarboxylation of isocitrate to generate α-ketoglutarate (α-KG). Cancer genetics revealed that IDH mutation was observed in several malignancies, including acute myeloid leukemia, chondrosarcoma, gliomas, and thyroid carcinoma. The most frequent mutations of IDH such as IDH1^R132H^ mutation exhibits decreased affinity for isocitrate, converting α-KG into D-2-hydroxyglutarate (D-2-HG)^26^. The accumulation of D-2-HG depletes carbohydrates from the Krebs cycle, leading to metabolic alterations. Moreover, D-2-HG blocks the activity of Ten-eleven translocation methyl cytosine dioxygenase (TET) and histone demethylases lysine-specific demethylase (KDM), resulting in CpG island hypermethylation and chromosomal instability^27^. IDH mutations are primarily detected in grade II and III gloma patients and serves as a favorable prognostic and therapeutic biomarker^28^. In both TCGA and CGGA datasets, we found that IDH mutant patients had lower risk scores than the wild type patients, indicating a favorable prognosis.

MGMT promoter methylation, and 1p19q codeletion were other two epigenetic alterations occurred in glioma patients. MGMT promoter methylation is associated with improved OS of GBM patients^29^. 1p19q codeletion is a combination of loss of the short arm chromosome 1 (1p) and the long arm of chromosome 19 (19q), 1p19q codeletion, MGMT promoter methylation and/or IDH1 mutation signified a better prognosis for glioma patients^30^. In this study, we found that patients with these molecular aberrations all had lower risk score than the wild type glioma patients. These results suggest that disulfidptosis‐related risk score model is positively correlated with these molecular aberrations and represents a valuable prognostic marker for glioma patients.

We further investigated the affected KEGG pathways and GO terms by differentially expressed genes between high-risk and low-risk glioma patients. Several sugar metabolism pathways and immune response biological processes were significantly enriched in high-risk patients. Disulfidptosis occurred under glucose starvation, and these results suggest that the three disulfidptosis-related gene may regulate disulfidptosis through sugar metabolisms. We also explored the correlation between the disulfidptosis-related signature and immune features. Several immunosuppressive molecules were consistently upregulated in high-risk group from all four datasets. The immune subtype analysis revealed that the ratio of C4 (Lymphocyte Depleted) subtype, which displays prominent M2 response, was higher in high-risk patients, and C4 immune subtype represents worse prognosis than C5 subtype^31^, further confirming the accuracy of the disulfidptosis‐related risk score model in predicting the prognosis and immune subtypes of glioma patients.

Currently, the immune checkpoint inhibitor therapy has improved patients’ survival among several cancers, and becomes one of the most promising cancer therapies^32^. PD-1 and PD-L1have been widely used in immune checkpoint inhibitor immunotherapy, and the results showed that both PD-1 and PD-L1 were increased in high-risk groups, and positively correlated with the disulfidptosis-related risk scores. These results suggest that disulfidptosis‐related risk signature can also predict the immune therapy response of glioma.

In conclusion, we developed a disulfidptosis‐related risk signature using LRPPRC, RPN1, and GYS1 genes. This disulfidptosis‐related risk signature is significantly correlated with prognosis, clinicopathological and immune features, and immune therapy response of glioma patients. The disulfidptosis‐related risk signature may represent an independent prognostic factor for glioma patients.

### Supplementary Information

Supplementary Tables.

## Data Availability

The datasets used during this study can be downloaded from public databases including TCGA, CGGA, and GEO: TCGA-LGG: https://portal.gdc.cancer.gov/projects/TCGA-LGG, TCGA-GBM: https://portal.gdc.cancer.gov/projects/TCGA-GBM. CGGA (mRNAseq_325): http://www.cgga.org.cn/download?file=download/20220620/CGGA.mRNAseq_325.Read_Counts-genes.20220620.txt.zip&type=mRNAseq_325_counts&time=20220620. CGGA_array (mRNA-array_301): http://www.cgga.org.cn/download?file=download/20200506/CGGA.mRNA_array_301_gene_level.20200506.txt.zip&type=mRNA_array_301_gene_level&time=20200506. GSE16011: https://www.ncbi.nlm.nih.gov/geo/query/acc.cgi?acc=GSE16011.

## Abbreviations

CNS: Central nervous system
LGG: Lower‐grade gliomas
GBM: Glioblastoma
IDH: Isocitrate dehydrogenase
MGMT: O-6-methylguanine-DNA methyltransferase
PCD: Programmed cell death
SLC7A11: Solute Carrier Family 7 Member 11
NADPH: Nicotinamide adenine dinucleotide phosphate hydrogen
TME: Tumor microenvironment
TCGA: The Cancer Genome Atlas
CGGA: Chinese Glioma Genome Atlas
GEO: Gene Expression Omnibus
OS: Overall survival

## Code availability

The R codes of this study was available at https://github.com/tjhwangxiong/Disulfidptosis_of_glioma.
