# Supplementary material for: The disulfidptosis-related signature predicts prognosis and immune features in glioma patients
Source: Sci Rep. 2023 Oct 20;13:17988. doi: 10.1038/s41598-023-45295-w (PMC10589205; doi:10.1038/s41598-023-45295-w)
Supplement: Supplementary file 1 — Supplementary Tables. [file 41598_2023_45295_MOESM1_ESM.docx]

**Table S1 univariable Cox regression analysis of disulfidptosis-related genes in glioma**

| **dataset** | **gene** | **coef** | **se** | **z** | **p** | **HR** | **HRCILL** | **HRCIUL** |
| --- | --- | --- | --- | --- | --- | --- | --- | --- |
| TCGA | LRPPRC | -0.97245 | 0.154972 | -6.27499 | 3.50E-10 | 0.378155 | 0.279098 | 0.512369 |
| TCGA | NCKAP1 | -0.65033 | 0.139987 | -4.64565 | 3.39E-06 | 0.521874 | 0.396651 | 0.68663 |
| TCGA | NDUFS1 | -1.27329 | 0.17496 | -7.27761 | 3.40E-13 | 0.279908 | 0.19865 | 0.394405 |
| TCGA | OXSM | 0.887869 | 0.150603 | 5.895441 | 3.74E-09 | 2.429946 | 1.808853 | 3.264298 |
| TCGA | RPN1 | 1.851783 | 0.128396 | 14.42248 | 0 | 6.371171 | 4.953689 | 8.194261 |
| TCGA | SLC7A11 | -0.24388 | 0.074503 | -3.27335 | 0.001063 | 0.783585 | 0.677125 | 0.906783 |
| TCGA | SLC3A2 | 0.527928 | 0.132047 | 3.998036 | 6.39E-05 | 1.695416 | 1.308813 | 2.196214 |
| TCGA | NUBPL | -2.53146 | 0.322376 | -7.85252 | 4.11E-15 | 0.079543 | 0.042286 | 0.149626 |
| TCGA | NDUFA11 | 0.309985 | 0.106557 | 2.909099 | 0.003625 | 1.363405 | 1.106428 | 1.680067 |
| TCGA | GYS1 | 1.090939 | 0.093631 | 11.65151 | 0 | 2.977067 | 2.477936 | 3.576739 |
| CGGA_array | GYS1 | 0.659525 | 0.139849 | 4.715987 | 2.41E-06 | 1.933874 | 1.470242 | 2.543711 |
| CGGA_array | LRPPRC | -0.51963 | 0.128225 | -4.05245 | 5.07E-05 | 0.594743 | 0.462577 | 0.764671 |
| CGGA_array | NCKAP1 | -0.2313 | 0.096348 | -2.40067 | 0.016365 | 0.793501 | 0.656955 | 0.958427 |
| CGGA_array | NDUFS1 | -0.50017 | 0.168393 | -2.97029 | 0.002975 | 0.606425 | 0.435954 | 0.843554 |
| CGGA_array | NUBPL | -0.29817 | 0.131224 | -2.27222 | 0.023073 | 0.742175 | 0.573863 | 0.959853 |
| CGGA_array | OXSM | 0.349882 | 0.177187 | 1.974646 | 0.048308 | 1.4189 | 1.002605 | 2.008046 |
| CGGA_array | RPN1 | 0.62445 | 0.13486 | 4.63036 | 3.65E-06 | 1.867219 | 1.433515 | 2.432137 |
| CGGA_array | SLC7A11 | -0.33683 | 0.069055 | -4.87764 | 1.07E-06 | 0.714033 | 0.623646 | 0.817519 |
| CGGA | GYS1 | 0.787454 | 0.092799 | 8.485553 | 0 | 2.197793 | 1.832297 | 2.636197 |
| CGGA | LRPPRC | -0.90328 | 0.187125 | -4.82713 | 1.39E-06 | 0.40524 | 0.280822 | 0.584781 |
| CGGA | NCKAP1 | -0.66049 | 0.137246 | -4.81242 | 1.49E-06 | 0.5166 | 0.394757 | 0.67605 |
| CGGA | NDUFA11 | 0.687408 | 0.16156 | 4.254827 | 2.09E-05 | 1.988555 | 1.448831 | 2.729338 |
| CGGA | NDUFS1 | -0.87117 | 0.147148 | -5.92035 | 3.21E-09 | 0.418463 | 0.313621 | 0.558354 |
| CGGA | NUBPL | -0.98852 | 0.212137 | -4.65981 | 3.17E-06 | 0.372127 | 0.245539 | 0.563979 |
| CGGA | OXSM | 0.444714 | 0.15535 | 2.862663 | 0.004201 | 1.560044 | 1.150543 | 2.115293 |
| CGGA | RPN1 | 1.683619 | 0.156657 | 10.74715 | 0 | 5.385007 | 3.961315 | 7.320371 |
| CGGA | SLC3A2 | 0.398874 | 0.130809 | 3.049289 | 0.002294 | 1.490145 | 1.153145 | 1.925632 |
| CGGA | SLC7A11 | -0.40042 | 0.080159 | -4.99532 | 5.87E-07 | 0.67004 | 0.572624 | 0.784028 |
| GSE16011 | GYS1 | 0.745314 | 0.145939 | 5.107024 | 3.27E-07 | 2.107104 | 1.582932 | 2.804849 |
| GSE16011 | RPN1 | 0.61548 | 0.161048 | 3.821718 | 0.000133 | 1.850545 | 1.349632 | 2.537371 |
| GSE16011 | LRPPRC | -0.62325 | 0.221885 | -2.80889 | 0.004971 | 0.536198 | 0.347101 | 0.828313 |
| GSE16011 | NUBPL | -1.1972 | 0.257911 | -4.64192 | 3.45E-06 | 0.302038 | 0.182191 | 0.500721 |

**Table S2 KM survival analysis of disulfidptosis-related genes in glioma**

| Gene | TCGA | CGGA_array | CGGA | GSE16011 |
| --- | --- | --- | --- | --- |
| RPN1 | 0 | 2.21E-07 | 0 | 9.17E-06 |
| GYS1 | 5.55E-16 | 1.54E-06 | 4.44E-16 | 0.004909 |
| NDUFS1 | 2.89E-12 | 0.000291 | 1.33E-08 | 0.008046 |
| NUBPL | 6.27E-11 | 0.004501 | 3.80E-05 | 0.037361 |
| LRPPRC | 2.88E-10 | 0.025093 | 9.36E-05 | 0.044141 |
| NCKAP1 | 7.42E-08 | 2.21E-07 | 0.0002 | 9.17E-06 |
| SLC3A2 | 2.02E-05 | 1.54E-06 | 0.000906 | 0.004909 |
| OXSM | 0.001028 | 0.000291 | 0.000972 | 0.008046 |
| SLC7A11 | 0.005918 | 0.004501 | 0.002889 | 0.037361 |
| NDUFA11 | 0.007184 | 0.025093 | 0.034694 | 0.044141 |
